# One-year clinical outcomes in patients with renal insufficiency after contemporary PCI: data from a multicenter registry

**DOI:** 10.1007/s00392-019-01575-y

**Published:** 2019-12-02

**Authors:** Sean S. Scholz, Lucas Lauder, Sebastian Ewen, Saarraaken Kulenthiran, Nikolaus Marx, Orazbek Sakhov, Floris Kauer, Adam Witkowski, Marco Vaglimigli, William Wijns, Bruno Scheller, Michael Böhm, Felix Mahfoud

**Affiliations:** 1 Klinik für Innere Medizin III, Kardiologie, Angiologie Und Internistische Intensivmedizin, Saarland University Hospital, Saarland University, Kirrberger Str. 1, Geb. 41, IMED, 66421 Homburg/Saar, Germany; 2 Medizinische Klinik I, Klinik für Kardiologie, Angiologie Und Internistische Intensivmedizin, RWTH Aachen University, Aachen, Germany; 3 Department of Interventional Cardiology, City Heart Center, Almaty, Kazakhstan; 4 Department of Cardiology, Albert Schweitzer Ziekenhius, Dordrecht, Netherlands; 5 Department of Interventional Cardiology and Angiology, Institute of Cardiology, Warsaw, Poland; 6 Universitätsklinik für Kardiologie, Universitätsspital Bern, Bern, Schweiz; 7 The Lambe Institute for Translational Medicine, National University of Ireland and Saolta University Healthcare Group, Galway, Ireland; 8 Institute for Medical Engineering and Science, Massachusetts Institute of Technology, Cambridge, MA USA

**Keywords:** Drug-eluting stent, Chronic kidney disease, Hemodialysis, Registry, End-stage renal disease

## Abstract

**Background:**

Chronic kidney disease (CKD) is highly prevalent in patients with coronary artery disease (CAD).

**Objective:**

The outcome following revascularization using contemporary technologies (new-generation abluminal sirolimus-eluting stents with thin struts) in patients with CKD (i.e., glomerular filtration rate of < 60 mL/min/1.73m^2^) and in patients with hemodialysis (HD) is unknown.

**Methods:**

e-Ultimaster is a prospective, single-arm, multi-center registry with clinical follow-up at 3 months and 1 year.

**Results:**

A total of 19,475 patients were enrolled, including 1466 patients with CKD, with 167 undergoing HD. Patients with CKD had a higher prevalence of overall comorbidities, multiple/small vessel disease (≤ 2.75 mm), bifurcation lesions, and more often left main artery treatments (all *p* < 0.0001) when compared with patients with normal renal function (reference). CKD patients had a higher risk of target lesion failure (unadjusted OR, 2.51 [95% CI 2.04–3.08]), target vessel failure (OR, 2.44 [95% CI 2.01–2.96]), patient-oriented composite end point (OR, 2.19 [95% CI 1.87–2.56]), and major adverse cardiovascular events (OR, 2.34 [95% CI 1.93–2.83, *p* for all < 0.0001]) as reference. The rates of target lesion revascularization (OR, 1.17 [95% CI 0.79–1.73], *p* = 0.44) were not different. Bleeding complications were more frequently observed in CKD than in the reference (all *p* < 0.0001).

**Conclusion:**

In this worldwide registry, CKD patients presented with more comorbidities and more complex lesions when compared with the reference population. They experienced higher rate of adverse events at 1-year follow-up.

**Graphic abstract:**

**Figure:**
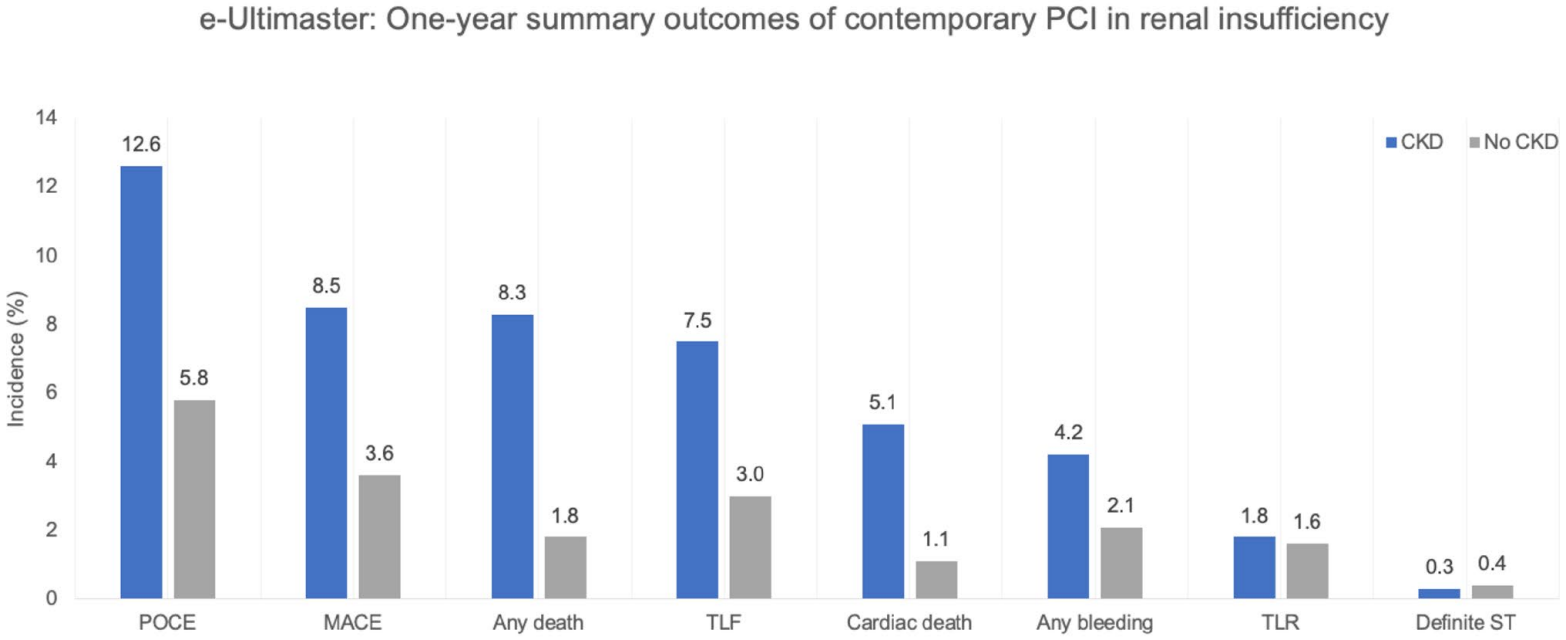
One-year summary outcomes of contemporary PCI in renal insufficiency. *CKD* chronic kidney disease, *POCE* patient oriented composite endpoint, *MACE* major adverse cardiovascular events, *TLF* target lesion failure, *TLR* target lesion revascularization, *ST* stent thrombosis

**Electronic supplementary material:**

The online version of this article (10.1007/s00392-019-01575-y) contains supplementary material, which is available to authorized users.

## Introduction

Chronic kidney disease (CKD) is highly prevalent, affecting more than 1.5 million patients in Europe and the USA and represents one of the most frequent comorbidities in patients with coronary artery disease (CAD) [1–4]. There is a linear relationship between cardiovascular mortality and impaired glomerular filtration rate (GFR) [5–8]. Severe and diffuse CAD is prevalent in patients with CKD [5–10]. In those patients, revascularization options include coronary artery bypass grafting (CABG) and percutaneous coronary intervention (PCI) [11–13]. Adverse event rates following PCI and CABG were significantly higher in CKD patients when compared to patients with normal renal function [5–8, 14–16]. Moreover, these patients differ from the general population concerning symptoms of acute cardiac events, poor access sites, complex vascular lesions, and a higher rate of complications [8, 13–18]. However, patients with CKD are under-represented in clinical studies on revascularization, and, thus, knowledge on performance of PCI using newest-generation drug-eluting stents (DES) is limited [9, 13]. We aimed at evaluating the outcomes following revascularization using contemporary technologies (abluminal sirolimus-eluting stents with thin struts) in patients with CKD (defined as an estimated glomerular filtration rate of < 60 mL/min/1.73m^2^) including a subgroup of patients undergoing hemodialysis (HD) in the prospective, single-arm, multi-center, international e-Ultimaster registry. CKD patients were compared with patients with normal kidney function (no CKD) undergoing sirolimus-eluting stent implantation.

## Methods

**Fig. 1:**
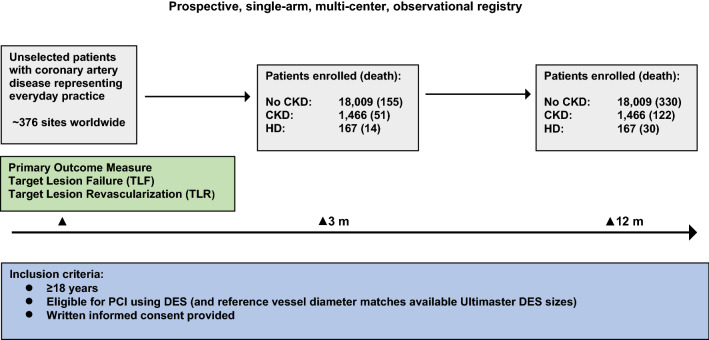
Prospective, single-arm, multi-center, observational registry. *CKD* chronic kidney disease, *HD* hemodialysis, *PCI* percutaneous coronary intervention, *DES* drug-eluting stent, *M* month

We analyzed the results of e-Ultimaster (NCT 02188355), a prospective, single-arm, multi-center, international registry with clinical follow-up at 3 months and 1 year (Fig. 1). This study sought to validate the safety and efficacy of an abluminal, sirolimus-coated stent with thin struts (Ultimaster DES) in unselected patients representing everyday practice. Secondary objectives were to evaluate the utilization of DES and the detection of rare events in a representative high-risk patient population such as patients with CKD (eGFR < 60 mL/min/1.73 m^2^). The predefined objective was to enroll a representative population of patients (≥ 400) with impaired renal function. Additionally, we aimed to identify predictors of major adverse events, to assess access site utilization, vascular complications, procedural particularities, the duration of dual antiplatelet therapy (DAPT), and overall performance of the newest-generation DES (Ultimaster) across different patient and lesion subsets. The primary outcome measure was target lesion failure (TLF) defined as a composite of cardiac death, target vessel-related myocardial infarction (MI), and clinically driven target lesion revascularization (TLR) at 1 year. Patient-oriented composite end point (POCE) was defined as any cause of mortality, any MI or any coronary revascularization. Target vessel failure rate (TVF) was defined as cardiac death, target vessel-related MI, and target vessel revascularization. Additionally, major adverse cardiac events (MACE: cardiac death, any MI, clinically driven TVR, and emergent CABG) were documented. Stent thrombosis was documented and defined according to the Academic Research Consortium definitions. An independent clinical event committee reviewed and adjudicated all end point-related serious adverse events. Inclusion criteria were age ≥ 18 years, eligibility for PCI using DES, reference vessel diameter matches, available Ultimaster DES sizes, and written informed consent. The registry was conducted in accordance with the Declaration of Helsinki and country-specific regulatory requirements. All patients signed informed consent form, reviewed and approved by the institutional review board/ethics committee of each participating center. Patients with CKD were automatically allocated to this sub-study (Fig. 1). The data were collected using Electronic CRF (e-Capture). Patients were enrolled between October 2014 and June 2018 and follow-up is currently still ongoing. The current analysis includes all patients enrolled between October 2014 and November 2016, who had a 1-year follow-up visit completed or had died by the date of census (30 November 2016).

### Statistical analyses

Patients’ demographics, comorbidities, medical history, target lesion characteristics, and procedural characteristics are summarized with mean, standard deviation for continuous variables, and with frequencies and percentages for discrete variables (Table 1, 2, 3, 4, supplement Table 1). All variables were tested for normal distribution with the Kolmogorov–Smirnov test. For non-normally distributed variables, the Wilcoxon-signed rank test was used. Fisher´s exact test or Chi-squared tests were used for categorical variables, as appropriate. Furthermore, medians with interquartile ranges (IQR) were reported where applicable. When appropriate, the multivariate logistic regression was used for dichotomous variables to adjust for the known risk and potential confounding factors identified with stepwise regression (multivariable *p* values thresholds to enter and stay in the model were 0.25 and 0.10, respectively), odds ratios and 95% confidence intervals. Variables considered for entry in the stepwise model include: age, gender, body mass index, smoking, diabetes, hypertension, hypercholesterolemia, renal failure, hemodialysis, family history of heart disease, history of MI, previous PCI, previous CABG, acute coronary syndrome, STEMI, multivessel disease, number of lesions identified, number of lesions treated, target vessel treated (right coronary artery, left main, left ascending coronary artery, circumflex artery, graft), type B2 and C lesions (according to classification of American College of Cardiology/American Heart Association/ [ACC/AHA]), bifurcation, moderate to severe calcification, chronic total occlusion, in-stent restenosis, ostial lesions, long lesions (≥ 25 mm), small vessels (≤ 2.75 mm), radial access, number of study stents implanted, total length of implanted study stents. Additionally, we included Kaplan–Meier estimates, Kaplan–Meier estimates adjusted for confounding using estimated propensity scores (“overlap weights” method), and hazard ratios for comparison of survival curves using a log-rank test where appropriate [19]. Patients were matched for lesion type C, number of lesions treated, number of study stents implanted, bifurcation, acute coronary syndrome, lesion type B2, previous percutaneous coronary intervention, ST elevated MI pre-procedure, number of lesions detected, use of intravascular ultrasound (IVUS)/coronary optical frequency domain imaging (OFDI), current smoker, diabetes mellitus, and age using the Xie, Liu method to control for possible confounding from these prognostic factors [19].

**Table 1:** Baseline patient characteristics

Baseline characteristics	CKD *N* = 1466	No CKD *N* = 18,009	*p* value
Age, years (IQR)	72.5 (64.0 to 79. 0)	64.0 (56.0 to 72.0)	< 0.0001
Male, % (*n*)	72.2 (1058/1466)	76.9 (13,849/18,009)	< 0.0001
Body mass index, kg/m^2^ (IQR)	27.2 (24.3 to 30.5)	27.2 (24.6 to 30.2)	0.82
Left ventricular ejection fraction, % (IQR)	55.0 (43.0 to 60.0)	56.0 (49.0 to 62.0)	< 0.0001
Diabetes, % (*n*)	48.9 (715/1466)	26.5 (4770/18,008)	< 0.0001
Hypertension, % (*n*)	83.9 (1206/1438)	62.2 (10,706/17,205)	< 0.0001
Hypercholesterolemia, % (*n*)	66.7 (949/1423)	55.9 (9609/17,185)	< 0.0001
Current smoker, % (*n*)	11.6 (156/1347)	24.3 (4055/16,716)	< 0.0001
Family history of heart disease, % (*n*)	17.8 (193/1083)	28.1 (3559/12,691)	< 0.0001
Previous CABG, % (*n*)	10.5 (151/1440)	5.6 (969/17,365)	< 0.0001
Previous PCI, % (*n*)	34.0 (491/1444)	24.9 (4344/17,456)	< 0.0001
Previous MI, % (*n*)	30.4 (436/1433)	21.5 (3749/17,461)	< 0.0001
Angina status before procedure			
Acute coronary syndrome, % (*n*)	50.5 (739/1463)	56.2 (10,114/17,998)	< 0.0001
NSTEMI, % (*n*)	28.8 (421/1463)	24.5 (4405/17,998)	0.0003
STEMI, % (*n*)	11.6 (169/1463)	20.7 (3724/17,998)	< 0.0001
Unstable angina, % (*n*)	10.2 (149/1463)	11.0 (1984/17,998)	0.34
Silent ischemia, % (*n*)	12.7 (185/1463)	8.6 (1541/17,998)	< 0.0001

Values are presented as mean ± standard deviation (SD), or %, or median with interquartile ranges (IQR 1–3)

*PCI* percutaneous coronary intervention, *CABG* coronary artery bypass graft, *MI* myocardial infarction, *CKD* chronic kidney disease, *(N)STEMI* (non-)ST elevated myocardial infarction, *N* number of patients

**Table 2:** Procedural details and differences

Peri-procedural details	CKD *N* = 1466	No CKD *N* = 18,009	*p* value
Number of vessels diseased PP, *N*	1.83 ± 0.8	1.62 ± 0.8	< 0.0001
Multiple vessels diseased PP, % (*n*)	57.6 (845/1466)	45.9 (8252/18,003)	< 0.0001
Multiple vessels treated PP, % (*n*)	17.3 (254/1465)	16.6 (2987/18,005)	0.47
Small vessels (≤2.75 mm) PP, % (*n*)	49.2 (718/1460)	43.9 (7878/17,941)	0.0001
Mean number of lesions identified PP, *N* (IQR)	2.0 (1.0 to 3.0)	1.0 (1.0 to 2.0)	< 0.0001
Mean number of lesions treated PP, *N* (IQR)	1.0 (1.0 to 2.0)	1.0 (1.0 to 2.0)	0.30
Left main artery treated PP, % (*n*)	6.6 (97/1465)	2.9 (515/18,005)	< 0.0001
Bifurcation PP, % (*n*)	16.8 (243/1451)	12.9 (2297/17,870)	< 0.0001
Bypass graft treated PP, % (*n*)	2.4 (35/1465)	1.1 (199/18,005)	< 0.0001
Number of stents successfully implanted PP (IQR)	1.0 (1.0 to 2.0)	1.0 (1.0 to 2.0)	0.03
Number of stents successfully implanted PL (IQR)	1.0 (1.0 to 1.0)	1.0 (1.0 to 1.0)	0.94
Length of stents implanted, mm PL (IQR)	24.0 (18.0 to 33.0)	24.0 (15.0 to 33.0)	0.05
Lesion type (ACC/AHA classification)			
B2 PL, % (*n*)	34.7 (593/1709)	28.0 (5611/20,027)	< 0.0001
C PL, % (*n*)	26.2 (448/1709)	26.4 (5291/20,027)	0.75
< 3 mm from ostium PL, % (*n*)	8.3 (164/1974)	5.7 (1369/23,967)	< 0.0001
Severe or moderate calcification PL, % (*n*)	28.3 (558/1974)	17.1 (4086/23,967)	< 0.0001
Peri-procedural differences			
Femoral access site PP, % (*n*)	25.2 (369/1466)	18.5 (3323/18,005)	< 0.0001
Radial access site PP, % (*n*)	74.2 (1088/1466)	82.8 (14,899/18,005)	< 0.0001
Brachial access site PP, % (*n*)	1.8 (27/1466)	0.3 (62/18,005)	< 0.0001
Direct stenting PL, % (*n*)	30.4 (604/1999)	38.6 (9331/24,203)	< 0.0001
Balloon dilatation only PL, % (*n*)	1.6 (31/1999)	2.3 (555/24,203)	0.03
Balloon pre-dilatation PL, % (*n*)	68.4 (1368/1999)	59.7 (14,436/24,203)	< 0.0001
Balloon post-dilatation PL, % (*n*)	43.6 (872/1999)	40.6 (9823/24,203)	0.007
Thrombus aspiration PL, % (*n*)	3.3 (65/1999)	5.0 (1221/24,203)	0.0002
Cutting balloon PL, % (*n*)	2.5 (50/1999)	1.2 (298/24,203)	< 0.0001
Atherectomy PL, % (*n*)	2.2 (44/1999)	0.7 (171/24,203)	< 0.0001
Microcatheter PL, % (*n*)	4.8 (96/1999)	2.5 (598/24,203)	< 0.0001
IVUS PP, % (*n*)	13.1 (164/1248)	5.3 (764/14,385)	< 0.0001
OFDI PP, % (*n*)	4.9 (61/1248)	3.0 (436/14,385)	0.0007

Values are presented as mean ± standard deviation (SD), % or median with interquartile ranges (IQR 1–3)

*ACC/AHA* American College of Cardiology/American Heart Association, *CKD* chronic kidney disease, *IVUS* intravascular ultrasound, *OFDI* coronary optical frequency domain imaging, *PP* per patient, *PL* per lesion, *N* number

**Table 3:** End points, complications, and follow-up (1 year)

Endpoints	CKD *N* = 1466	No CKD *N* = 18,009	*p* value
Any death, % (*n*)	8.3 (121/1466)	1.8 (327/18,009)	< 0.0001
Cardiac death, % (*n*)	5.1 (74/1466)	1.1 (203/18,009)	< 0.0001
Any MI, % (*n*)	1.6 (23/1466)	1.1 (195/18,009)	0.07
Target vessel MI, % (*n*)	1.4 (20/1466)	0.9 (155/18,009)	0.06
Clinically driven TLR, % (*n*)	1.8 (27/1466)	1.6 (283/18,009)	0.45
Clinically driven TVR, % (*n*)	3.0 (44/1466)	2.2 (387/18,009)	0.04
Composite end points			
TLF, % (*n*)	7.5 (109/1466)	3.0 (534/18,009)	< 0.0001
TVF, % (*n*)	8.4 (123/1466)	3.4 (619/18,009)	< 0.0001
POCE, % (*n*)	12.6 (185/1466)	5.8 (1039/18,009)	< 0.0001
MACE, % (*n*)	8.5 (124/1466)	3.6 (654/18,009)	< 0.0001
Stent thrombosis			
Definite ST, % (*n*)	0.3 (5/1466)	0.4 (76/18,009)	0.83
Probable ST, % (*n*)	0.4 (6/1466)	0.2 (41/18,009)	0.16
Definite and probable ST, % (*n*)	0.8 (11/1466)	0.6 (115/18,009)	0.61
Complications (reported)			
Any bleeding, % (*n*)	4.2 (61/1466)	2.1 (384/18,009)	< 0.0001
Major bleeding, % (*n*)	1.4 (20/1466)	0.5 (97/18,009	0.0003
Minor bleeding, % (*n*)	2.9 (43/1466)	1.6 (289/18,009)	0.0002
Complication related to access site, % (*n*)	2.2 (32/1466)	1.1 (203/18,009)	0.001
Follow-up			
Days from procedure to discharge	3.2 ± 4.1	2.4 ± 2.9	< 0.0001
DAPT 3-month follow-up, % (*n*)	89.5 (1282/1433)	94.8 (16,823/17,748)	< 0.0001
DAPT 12-month follow-up, % (*n*)	60.9 (843/1384)	67.0 (11,898/17,747)	< 0.0001

*CKD* chronic kidney disease, *DAPT* dual antiplatelet therapy, *MACE* major adverse cardiac events (cardiac death, any MI, clinically driven TVR and emergent coronary artery bypass graft), *MI* myocardial infarction, *POCE* patient-oriented composite end point (all death, any MI, any coronary revascularization), *ST* stent thrombosis, *TLF* target lesion failure (cardiac death, target vessel MI, clinically driven TLR), *TLR* target lesion revascularization, *TVF* target vessel failure (cardiac death, target vessel MI, clinically driven TVR), *TVR* target vessel revascularization, *N* number of patients

**Table 4:** Summary results of patients on HD

Summary results	HD *N* = 167	CKD (no HD) *N* = 1299	*p* value
Age, years (IQR)	68.0 (61.0 to 75. 0)	73.0 (65.0 to 79.0)	< 0.0001
Male, % (*n*)	67.1 (112/167)	72.8 (946/1299)	0.12
Body mass index, kg/m2 (IQR)	26.0 (22.4 to 28.8)	27.4 (24.6.0 to 30.7)	< 0.0001
Diabetes, % (*n*)	63.5 (106/167)	46.9 (609/1299)	< 0.0001
Hypertension, % (*n*)	86.4 (140/162)	83.5 (1066/1276)	0.43
Hypercholesterolemia, % (*n*)	67.1 (108/161)	66.6 (841/1262)	0.99
Current smoker, % (*n*)	8.1 (12/149)	12.0 (144/1198)	0.18
Family history of heart disease, % (*n*)	11.5 (15/131)	18.7 (178/952)	0.05
Left ejection fraction, % (IQR)	53.0 (45.0 to 63.0)	55.0 (42.0 to 60.0)	0.46
Previous CABG, % (*n*)	7.3 (12/164)	10.9 (139/1276)	0.18
Previous PCI, % (*n*)	33.3 (55/165)	34.1 (436/1279)	0.93
Previous MI, % (*n*)	29.3 (48/164)	30.6 (388/1269)	0.79
Peri-procedural differences			
Left main treated, % (*n*)	12.0 (20/167)	5.9 (77/1298)	0.007
Small vessels (≤2.75 mm), % (*n*)	52.1 (87/166)	48.8 (631/1294)	0.46
Multiple vessels diseased, % (*n*)	58.7 (98/167)	57.5 (747/1299)	0.80
Multiple vessels treated, % (*n*)	24.6 (41/167)	16.4 (213/1298)	0.01
Number of vessels diseased, *n*	1.80 ± 0.8	1.83 ± 0.8	0.63
Femoral access site, % (*n*)	53.9 (90/167)	21.5 (279/1299)	< 0.0001
Radial access site, % (*n*)	40.1 (67/167)	78.6 (1021/1299)	< 0.0001
Brachial access site, % (*n*)	6.6 (11/167)	1.2 (16/1299)	< 0.0001
End points at 1-year			
Any death, % (*n*)	16.2 (27/167)	7.3 (94/1299)	0.0005
Cardiac death, % (*n*)	7.8 (13/167)	4.8 (61/1299)	0.13
Non-cardiac death, % (*n*)	8.4 (14/167)	2.5 (33/1299)	0.0004
Target vessel MI, % (*n*)	0.6 (1/167)	1.5 (22/1299)	0.72
Clinically driven TLR, % (*n*)	3.6 (6/167)	1.7 (21/1299)	0.12
Clinically driven TVR, % (*n*)	5.4 (9/167)	2.7 (35/1299)	0.09
Composite endpoints			
TLF, % (*n*)	11.4 (19/167)	6.9 (90/1299)	0.06
TVF, % (*n*)	12.6 (21/167)	7.9 (102/1299)	0.05
POCE, % (*n*)	21.0 (35/167)	11.6 (150/1299)	0.001
MACE, % (*n*)	12.6 (21/167)	7.9 (103/1299)	0.05
Stent thrombosis			
Definite ST, % (*n*)	0.6 (1/167)	0.3 (4/1299)	0.45
Probable ST, % (*n*)	0.6 (1/167)	0.4 (5/1299)	0.52
Possible ST, % (*n*)	3.0 (5/167)	2.3 (30/1299)	0.59
Complications			
Major bleeding, % (*n*)	3.0 (5/167)	1.2 (15/1299)	0.07
Minor bleeding, % (*n*)	2.4 (4/167)	3.0 (39/1299)	0.81
Complication related to access site, % (*n*)	0.6 (1/167)	2.4 (31/1299)	0.17
Follow-up			
DAPT 3-month follow-up, % (*n*)	81.7 (134/164)	90.5 (1148/1269)	0.002
DAPT 1-year follow-up, % (*n*)	67.1 (100/149)	60.2 (743/1235)	0.08

Values are presented as mean ± standard deviation (SD), % or median with interquartile ranges (IQR 1–3)

*CKD* chronic kidney disease, *DAPT* dual antiplatelet therapy, *HD* hemodialysis, *MACE* major adverse cardiac events (cardiac death, any MI, clinically driven TVR and emergent coronary artery bypass graft), *MI* myocardial infarction, *POCE* patient-oriented composite endpoint (all death, any MI, any coronary revascularization), *ST* stent thrombosis, *TLF* target lesion failure (cardiac death, target vessel MI, clinically driven TLR), *TLR* target lesion revascularization, *TVF* target vessel failure (cardiac death, target vessel MI, clinically driven TVR), *TVR* target vessel revascularization, *CABG* coronary artery bypass graft, *PCI* percutaneous coronary intervention, *N* number of patients

## Results

### Patient characteristics

A total of 19,475 patients were eligible for inclusion (Fig. 1). Of these, 1,466 had CKD including 167 patients undergoing HD. Patients with CKD were older (CKD: 72.5 IQR: 64–79 vs. no-CKD: 64.0 IQR: 56–72, *p* < 0.0001), had a lower left ventricular ejection fraction (CKD: 55.0 IQR: 43–60 vs. no-CKD: 56.0 IQR: 49–62, *p* < 0.0001), and presented with more comorbidities including diabetes (CKD: 48.9% vs. no-CKD: 26.5%, *p* < 0.0001), hypertension (CKD: 83.9% vs. no-CKD: 62.2%, *p* < 0.0001), and hypercholesterolemia (CKD: 66.7% vs. no-CKD: 55.9%, *p* < 0.0001) compared with patients with normal kidney function (Table 1). Patients without CKD consisted of more than twice as many current smokers (CKD: 11.6% vs. no-CKD: 24.3%, *p* < 0.0001) and included a higher percentage with positive family history of heart disease (CKD: 17.8% vs. no-CKD: 28.1%, *p* < 0.0001). Of note, patients without CKD had a significantly lower number of previous CABG (CKD: 10.5% vs. no-CKD: 5.6%, *p* < 0.0001), PCI (CKD: 34.0% vs. no-CKD: 24.9%, *p* < 0.0001), and myocardial infarction (CKD: 30.4% vs. no-CKD: 21.5%, *p* < 0.0001). In addition, CKD patients had lower rates of acute coronary syndrome (CKD: 50.5% vs. no-CKD: 56.2%, *p* < 0.0001) and higher rates of silent ischemia at baseline (CKD: 12.7% vs. no-CKD: 8.6%, *p* < 0.0001).

### Procedural details

Patients with CKD had a higher average number of vessels diseased, lesions in total, and small vessel disease (≤ 2.75 mm; all *p* ≤ 0.0001), as summarized in Table 2. The lesions were more commonly located within 3 mm from the ostium (CKD: 8.3% vs. no-CKD: 5.7%, *p* < 0.0001), involved left main artery treatment (CKD: 6.6% vs. no-CKD: 2.9%, *p* < 0.0001), and more commonly affected bifurcations (CKD: 16.8% vs. no-CKD: 12.9%, *p* < 0.0001). In-stent restenosis of DES were significantly more often observed among patients with CKD (CKD: 4.5% vs. no-CKD: 3.0%, *p* = 0.002) with no difference in in-stent restenosis of bare-metal stents (BMS; CKD: 1.9% vs. no-CKD: 1.8%, *p* = 0.84). Additionally, the total length of successfully implanted stents was slightly higher in CKD (per lesion; CKD: 27.0 ± 16 mm vs. no-CKD 25.8 ± 14, *p* = 0.002). Further, procedures in CKD patients were more complex when compared with the reference population (less frequent direct stenting; CKD: 30.4% vs. no-CKD: 38.6%, *p* < 0.0001; balloon dilatation only; CKD: 1.6% vs. no-CKD: 2.3%, *p* = 0.03; thrombus aspiration; CKD: 3.3% vs. no-CKD: 5.0%, *p* = 0.0002). Support devices (such as balloon pre-dilatation, post-dilatation, cutting balloon, atherectomy, microcatheters) and peri-interventional imaging (IVUS/ OFDI) were more commonly used in patients with CKD (Table 2). Although, radial access was used in the majority of cases in both groups (CKD: 74.2% vs. no-CKD: 82.8%, *p* < 0.0001), it was significantly less utilized in patients with CKD. In contrast, femoral (CKD: 25.2% vs. no-CKD: 18.5%, *p* < 0.0001) and brachial access (CKD: 1.8% vs. no-CKD: 0.34%, *p* < 0.0001) were more frequently used in CKD.

### Safety and efficacy

**Fig. 2:**
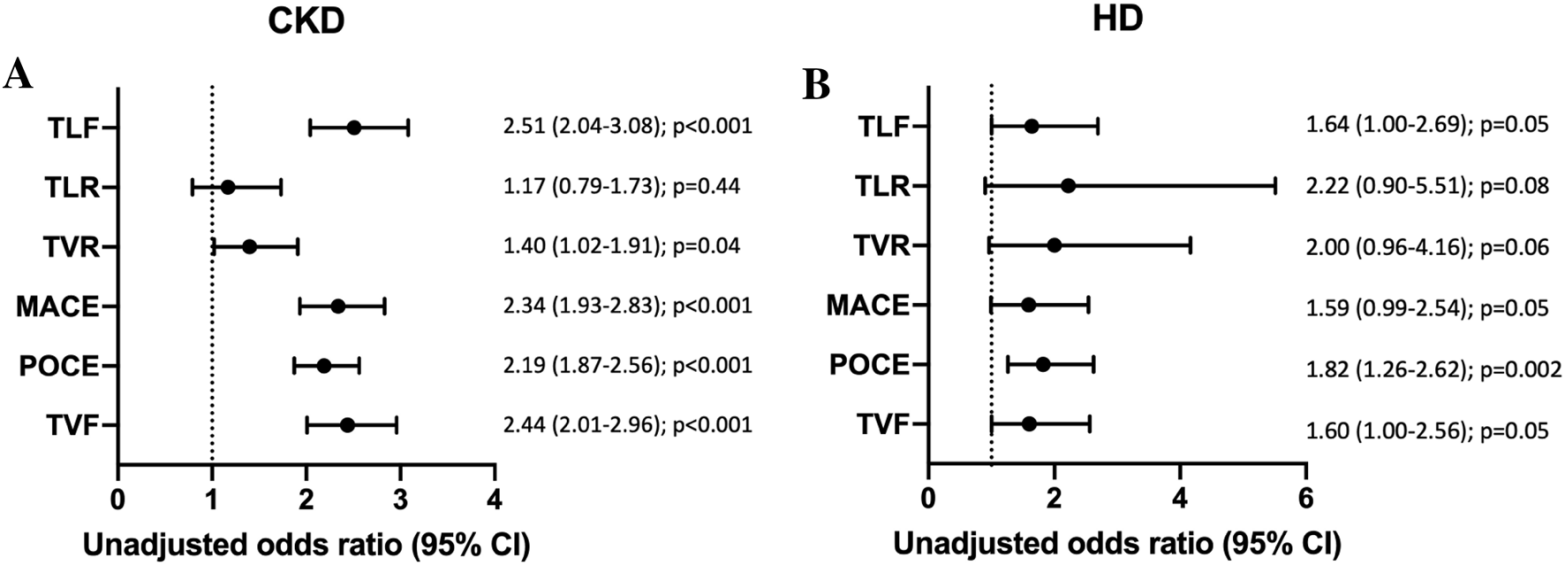
One-year unadjusted odds ratios for outcomes in patients with chronic kidney disease (CKD) compared with the reference population (**a**) and 1-year unadjusted odds ratios for outcomes in patients with hemodialysis (HD) compared to patients with CKD (**b**). *TLF* target lesion failure, *TLR* target lesion revascularization, *TVR* target vessel revascularization, *MACE* (cardiac death, any MI, clinically driven TVR, and emergent CABG): major adverse cardiovascular events, *POCE* patient-oriented composite end point (all death, any MI, any coronary revascularization), *TVF* target vessel failure rates, *CI* confidence interval

**Fig. 3:**
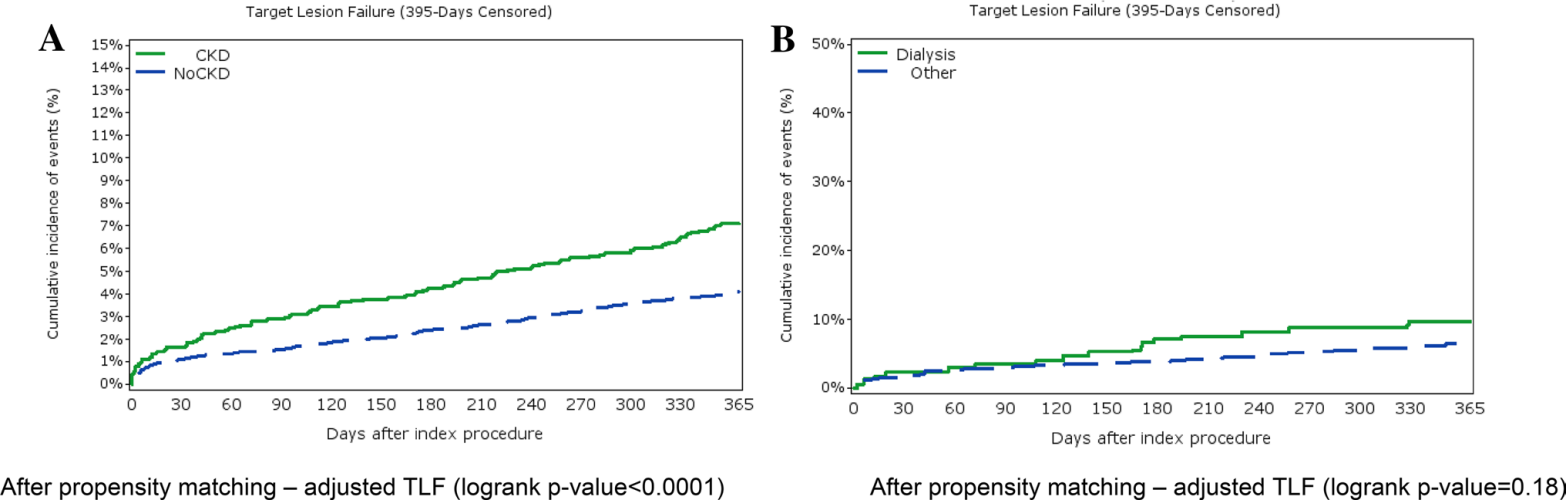
Kaplan–Meier estimates adjusted for confounding using estimated propensity scores (Xie, Liu method); cumulative events for target lesion failure; chronic kidney disease (CKD) versus reference population, **a**, and hemodialysis patients versus reference population, **b**; *TLF* target lesion failure

**Fig. 4:**
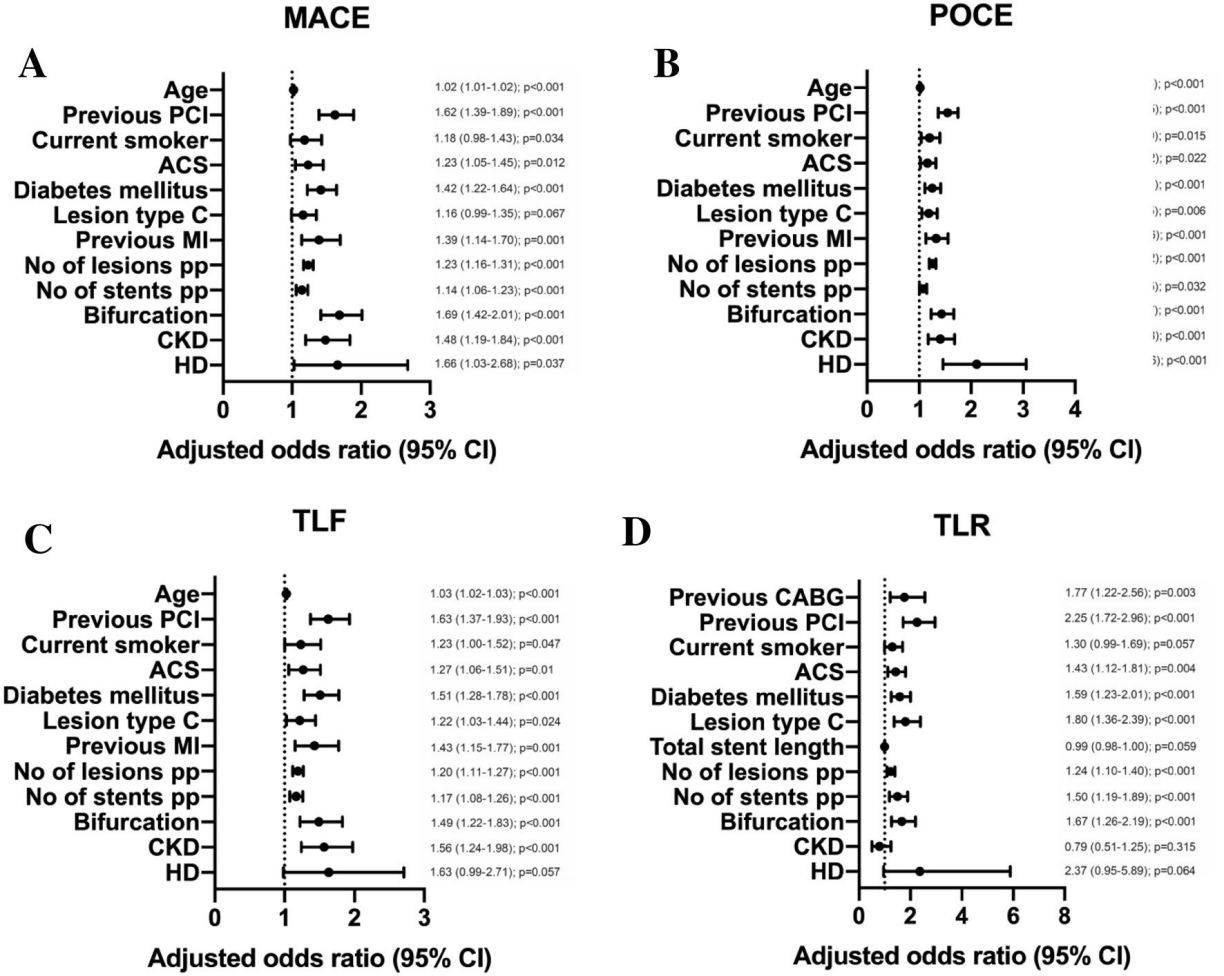
One-year adjusted odds ratios for major adverse cardiovascular events (MACE: cardiac death, any MI, clinically driven TVR, and emergent coronary artery bypass graft, **a** patient-oriented composite end point (POCE: all death, any MI, any coronary revascularization, **b** target lesion failure (TLF: cardiac death, target vessel MI, clinically driven TLR, **c** and target lesion revascularization TLR, **d** yes versus no; *CKD* chronic kidney disease, *HD* hemodialysis, *PCI* percutaneous coronary intervention, *No* number, *MI* ST elevated myocardial infarction, *CI* confidence interval, *ACS* acute coronary syndrome, *PP* per patient, *CABG* coronary artery bypass graft

Safety and efficacy data were available for 19,475 patients at baseline and 12-month follow-up. Reported complications related to access site (CKD: 2.2% vs. no-CKD: 1.1%, *p* = 0.001), minor bleeding (CKD: 3.1% vs. no-CKD: 1.6%, *p* = 0.0002), and major bleeding (CKD: 1.4% vs. no-CKD: 0.5%, *p* = 0.0003) occurred more commonly among patients with CKD (Table 3). However, CKD patients had a significantly higher percentage of patients treated with oral anticoagulation at baseline (CKD: 11.1% vs. no-CKD: 4.0%, *p* < 0.001). The primary outcome measure TLF (composite of cardiac death, target vessel-related MI, and clinically driven TLR) occurred more often in the CKD group (unadjusted OR, 2.51 [95% CI 2.04–3.08] *p* < 0.0001; Fig. 2a). Adjusted Kaplan–Meier estimates on TLF indicate significantly more events in CKD patients as depicted in Fig. 3. Furthermore, patients with CKD were at higher risk for TVF (OR, 2.44 [95% CI 2.01–2.96]; *p* < 0.0001), POCE (OR, 2.19 [95% CI 1.87–2.56] *p* < 0.0001), and MACE (OR, 2.34 [95% CI 1.93–2.83] *p* < 0.0001). In contrast, the risk for TLR (OR, 1.17 [95% CI 0.79–1.73] *p* = 0.44) did not differ significantly between patients with and without CKD (Fig. 2a). Unadjusted Kaplan–Meier estimates on cumulative incidence of TLF, clinically driven TLR, TVF, and POCE are provided in supplement Fig. 1. The 1-year risk of TLF was significantly increased by age, body mass index, diabetes, smoking status, previous PCI, ACS, number of lesions, target vessel location, bifurcation involvement, type C lesions, number of stents implanted, CKD, and hemodialysis. One-year adjusted odds ratios for MACE, POCE, TLF, and TLR are presented in Fig. 4.

### Subgroup of HD patients

Patients with CKD on HD (11.4% of all patients with CKD) were younger (*p* < 0.0001), had a lower body mass index (*p* < 0.0001), a higher prevalence of diabetes (*p* < 0.0001), and less frequently a family history of heart diseases (*p* = 0.05) compared with patients with CKD not requiring HD (Table 4). In CKD patients on HD, left main arteries were more frequently treated (HD: 12.0% vs. CKD: 5.9%, *p* = 0.007). Although multiple vessels were not more commonly diseased (HD: 58.7% vs. CKD: 57.7%, *p* = 0.80), they were more likely to be treated in HD patients (HD: 24.6% vs. CKD: 16.4%, *p* = 0.01). Debulking strategies were commonly utilized in HD patients, such as balloon pre-dilatation, atherectomy, microcatheters, and intravascular ultrasound. The preferred access site in HD patients was femoral access, followed by radial or brachial, which is different compared with the CKD population (all; *p* < 0.0001; Table 4). Access site complications were comparable between the two groups. At 3 months, DAPT rates were significantly lower in patients with CKD on HD compared with non-HD patients with no significant difference at 1-year follow-up (Table 4). All-cause death occurred more commonly in patients with HD (HD: 16.2% vs. CKD: 7.3%, *p* = 0.0005), with no significant difference in cardiac death (HD: 7.8% vs. CKD: 4.8%, *p* = 0.13), but a difference in non-cardiac death (HD: 8.4% vs. CKD: 2.5%, *p* = 0.0004), respectively. In comparison to the included CKD population, HD further increased the risk for the predefined end points TLF (unadjusted OR, 1.64 [95% CI 1.00–2.69] *p* = 0.049,TLR (OR, 2.22 [95% CI 0.90–5.51] *p* = 0.08, TVR (OR, 2.00 [95% CI 0.96–4.16] *p* = 0.06**,** MACE (OR, 1.59 [95% CI 0.99–2.54] *p* = 0.05), POCE (OR, 1.82 [95% CI 1.26–2.62] *p* = 0.002), and TVF (OR, 1.60 [95% CI 1.00–2.56]; *p* = 0.049) (Fig. 2b).

## Discussion

In this large, contemporary, international, real-world registry, patients with CKD undergoing new-generation DES implantation have a significantly higher risk of 1-year adverse events including TLF, defined as the composite of cardiac death, target vessel-related MI, clinically driven TLR, and more bleeding events. Patients with CKD had a higher prevalence of comorbidities and complex coronary lesions frequently requiring debulking strategies. Compared with other risk factors, the presence of CKD, and in particular need for HD, was one of the most impactful parameters in increasing the risk of TLF, MACE, and POCE.

Chronic kidney disease is a worldwide growing health issue, affecting more than 1.5 million patients in Europe and the USA and is associated with worse outcome [1, 2]. Cardiovascular disease, specifically CAD, is the leading cause of morbidity and mortality in patients with CKD. The optimal revascularization strategy is being debated and includes CABG and PCI, preferably with the use of DES in CKD [11–13, 20, 21]. While CABG was shown to be superior to PCI in patients with multivessel CAD and end-stage renal disease regarding long-term survival, PCI was superior to CABG in short-term survival, stroke, and repeat revascularization [11]. However, data on the long-term performance of new-generation DES indicate no significant difference between PCI and CABG in patients with CKD [11–13, 22–24]. Previous studies on revascularization have shown that patients with CKD after PCI and CABG are at increased risk of death and adverse events, correlating with the severity of renal insufficiency [23–25]. Herein, the 1-year event rates for patients with CKD were significantly higher when compared with the reference population. In contrast, the 1-year event rate for patients with preserved renal function was comparable to that in other recently published studies evaluating the safety and performance of new-generation DES indicating good performance of the Ultimaster stent. The prospective COMBO Stent registry, for instance, included 3614 patients and documented TLF in 3.9%, cardiac death in 1.6%, and definite stent thrombosis in 0.5% (versus 3.3%, 1.4%, and 0.4% herein) [25–30]. Although complication rates were relatively low in general, patients with CKD had a higher risk of major and minor bleeding as well as access site-related complications leading to prolonged hospitalization and higher rates of end points at discharge (supplement Table 1), which is in line with previous publications [28–32]. Bleeding occurred in 2.3% of all included patients, with twofold higher rates of minor and major bleeding in CKD. In this context, the choice of access site is of special importance. Interestingly, the rates of radial access were lower in CKD when compared with no-CKD patients (74% vs. 83%, *p* < 0.0001), implying that operators might have chosen different arterial accesses in CKD. One may speculate that this was due to an expectably higher complexity of PCI requiring debulking strategies, intravascular imaging, and maintaining radial arterial integrity to provide future dialysis vascular access. Of note, the use of radial access was associated with a 24% lower risk (OR 0.76, 95% CI 0.63–0.91, *p* = 0.003) of TLF (Fig. 4) and should also be considered as a bleeding avoidance strategy in patients with CKD.

The deleterious effect of CKD on the vasculature is reflected by significant anatomical and procedural differences (Tables 2 and 4). Patients with CKD have increased prevalence of medial calcification, which may impair response to PCI and pose several challenges [20–22]. This is supported by our findings showing a higher number of affected vessels, small vessel disease (≤ 2.75 mm), in-stent restenosis (primarily in DES), and severely calcified lesions, which were more commonly located in the left main artery. As a result, lesions in CKD patients were frequently treated using peri-interventional imaging (IVUS, OFDI) and debulking strategies, such as balloon pre- and post-dilatation, cutting balloon, atherectomy, and microcatheters. Although more lesions were identified in CKD patients, the number of treated lesions did not differ when compared with the reference population, which might be another indicator for the higher complexity in CKD patients leading to a higher rate of non-complete revascularization.

There is a linear relationship between cardiovascular mortality and decreasing GFR with the highest cardiovascular morbidity and mortality in patients on HD [5–8]. Patients with CKD on HD exhibit a high risk for cardiovascular events as well as an altered coagulation with both increased thrombotic and bleeding risks, which may be caused by denser clot structures [33]. Interestingly, HD patients were younger compared with CKD non-HD patients and, apart from a higher prevalence of diabetes (64%), did not have significantly more comorbidities. Patients with CKD on HD had higher rates of all-cause mortality (including non-cardiac death) and TLF at 1 year compared with patients with CKD not requiring HD (Table 4). Of note, data on the 3-month and 1-year follow-up indicate a lower percentage of patients on DAPT in patients with CKD when compared to the reference population. This might be associated with higher bleeding rates in CKD suspected by patients and physicians, which were subsequently documented in this registry, although with a higher rate of patients on oral anticoagulation in patients with CKD in this registry.

## Limitations

It is recognized that registry-based studies have limitations. Follow-up procedures were not standardized and, thus, may have influenced the reporting of safety and adverse events. This may have led to underreporting of adverse events, which may have occurred, due to the discretion of the investigator. The characteristics of the patients included in this study were representative of patients with CKD in everyday practice. However, the results could be influenced by confounders in patient characteristics such as age and comorbidities. Furthermore, there may be biological heterogeneity, e.g., due to different disease severity regarding categorical variables, different glomerular filtration rates within the groups, and underlying causes of CKD within the groups. Also, adherence to medication is often dynamic, and rigorous assessment with toxicological screening and type of DAPT was not reported.

## Conclusion

In this large cohort of all-comer patients undergoing new-generation DES implantation, CKD and in particular HD were associated with a higher risk of adverse events including TLF when compared with the reference population. Although higher rates of adverse events were observed, low stent thrombosis rates indicate good performance in this high-risk patient population. Additional research on this topic preferably in randomized controlled trials is required to validate these findings.

## Clinical perspectives

CKD is a common comorbidity in everyday practice and patients with CAD. Moreover, these patients present with more comorbidities and complex lesions when compared with the reference population. Therefore, a high level of expertise is necessary to assure sufficient treatment. Randomized controlled trials are needed to validate these findings.

## Electronic supplementary material

Below is the link to the electronic supplementary material.

Supplementary file1 (DOCX 132 kb)
