# Correction to: One‑year clinical outcomes in patients with renal insuffciency after contemporary PCI: data from a multicenter registry

**DOI:** 10.1007/s00392-020-01600-5

**Published:** 2020-02-25

**Authors:** Sean S. Scholz, Lucas Lauder, Sebastian Ewen, Saarraaken Kulenthiran, Nikolaus Marx, Orazbek Sakhov, Floris Kauer, Adam Witkowski, Marco Vaglimigli, William Wijns, Bruno Scheller, Michael Böhm, Felix Mahfoud

**Affiliations:** 1 Klinik für Innere Medizin III, Kardiologie, Angiologie Und Internistische Intensivmedizin, Saarland University Hospital, Saarland University, Kirrberger Str. 1, Geb. 41, IMED, 66421 Homburg/Saar, Germany; 2 Medizinische Klinik I, Klinik für Kardiologie, Angiologie Und Internistische Intensivmedizin, RWTH Aachen University, Aachen, Germany; 3 Department of Interventional Cardiology, City Heart Center, Almaty, Kazakhstan; 4 Department of Cardiology, Albert Schweitzer Ziekenhius, Dordrecht, Netherlands; 5 Department of Interventional Cardiology and Angiology, Institute of Cardiology, Warsaw, Poland; 6 Universitätsklinik für Kardiologie, Universitätsspital Bern, Bern, Schweiz; 7 The Lambe Institute for Translational Medicine, National University of Ireland and Saolta University Healthcare Group, Galway, Ireland; 8 Institute for Medical Engineering and Science, Massachusetts Institute of Technology, Cambridge, MA USA

## Correction to: Clinical Research in Cardiology 10.1007/s00392-019-01575-y

The original version of this article unfortunately contained a mistake. The given name and family name of the fourth author Saarraaken Kulenthiran were switched in the original publication.

The original article has been corrected.

**Open Access** This article is distributed under the terms of the Creative Commons Attribution 4.0 International License (http://creativecommons.org/licenses/by/4.0/), which permits unrestricted use, distribution, and reproduction in any medium, provided you give appropriate credit to the original author(s) and the source, provide a link to the Creative Commons license, and indicate if changes were made.

