# Supplementary material for: One-year clinical outcomes in patients with renal insufficiency after contemporary PCI: data from a multicenter registry
Source: Clin Res Cardiol. 2019 Dec 2;109(7):845–56. doi: 10.1007/s00392-019-01575-y (PMC7308257; doi:10.1007/s00392-019-01575-y)
Supplement: Supplementary file 1 — Supplementary file1 (DOCX 132 kb) [file 392_2019_1575_MOESM1_ESM.docx]

**Supplement**

Supplement table 1. Endpoints at discharge

| Endpoints at discharge | CKD  N=1466 | No CKD  N=18009 | p-Value |
| --- | --- | --- | --- |
| Any death, % (n) | 1.02% (15/1466) | 0.30% (54/18009) | <0.001 |
| Any MI, % (n) | 0.27% (4/1466) | 0.33% (60/18009) | 0.70 |
| Target-vessel MI, % (n) | 0.27% (4/1466) | 0.32% (57/18009) | 0.77 |
| Clinically-driven TLR, % (n) | 0.07% (1/1466) | 0.19% (34/18009) | 0.29 |
| Clinically-driven TVR, % (n) | 0.07% (1/1466) | 0.23% (42/18009) | 0.20 |
| Composite endpoints |  |  |  |
| TLF, % (n) | 1.16% (17/1466) | 0.57% (103/18009) | 0.006 |
| TVF, % (n) | 1.16% (17/1466) | 0.59% (107/18009) | 0.009 |
| POCE, % (n) | 1.30% (19/1466) | 0.74% (134/18009) | 0.02 |
| MACE, % (n) | 1.16% (17/1466) | 0.62% (111/18009) | 0.01 |
| Stent thrombosis |  |  |  |
| Definite ST, % (n) | 0.07% (1/1466) | 0.17% (31/18009) | 0.34 |
| Probable ST, % (n) | 0.20% (3/1466) | 0.05% (9/18009) | 0.02 |
| Definite and probable ST, % (n) | 0.27% (4/1466) | 0.22% (39/18009) | 0.66 |
| Complications (reported) |  |  |  |
| Any bleeding, % (n) | 0.82% (12/1466) | 0.45% (81/18009) | 0.049 |
| Major bleeding, % (n) | 0.34% (5/1466) | 0.09% (17/18009) | 0.007 |
| Minor bleeding, % (n) | 0.48% (7/1466) | 0.36% (64/18009) | 0.46 |
| Complication related to access site, % (n) | 1.98% (29/1466) | 1.04% (187/18009) | 0.001 |

CKD: chronic kidney disease; MACE: major adverse cardiac events (cardiac death, any MI, clinically-driven TVR and emergent coronary artery bypass graft); MI: myocardial infarction; POCE: patient oriented composite endpoint (all death, any MI, any coronary revascularization); ST: stent thrombosis; TLF: target lesion failure (cardiac death, target-vessel MI, clinically-driven TLR); TLR: target lesion revascularization; TVF: target vessel failure (cardiac death, target-vessel MI, clinically-driven TVR); TVR: target vessel revascularization; N: number of patients


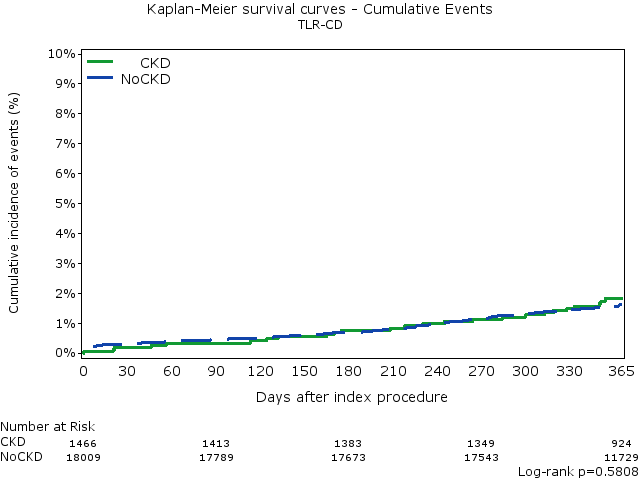

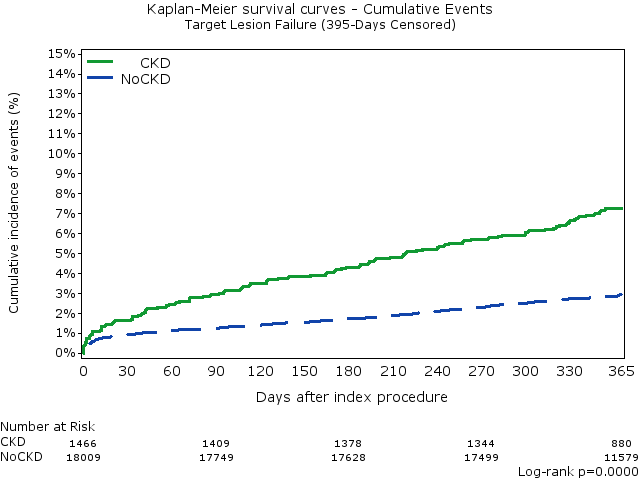
A. B.


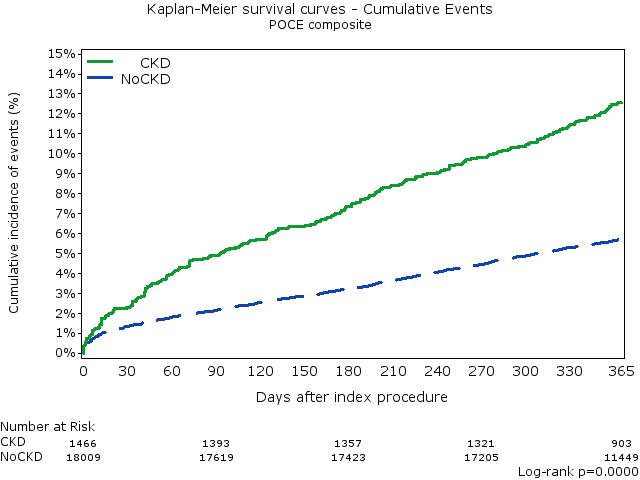

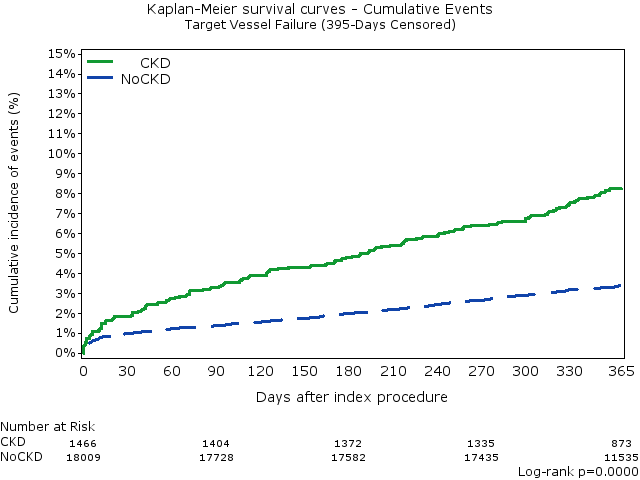
C. D.

**Supplement figure 1.** Kaplan-Meier curves (unadjusted); Cumulative incidence of target lesion failure, A), Clinically driven target lesion revascularization, B), Target vessel failure, C) and POCE, D); Chronic kidney disease (CKD) versus reference population
